# Baculovirus-assisted Reovirus Infection in Monolayer and Spheroid Cultures of Glioma cells

**DOI:** 10.1038/s41598-017-17709-z

**Published:** 2017-12-15

**Authors:** Iris J. C. Dautzenberg, Sanne K. van den Hengel, Jeroen de Vrij, Lars Ravesloot, Steve J. Cramer, Saw-See Hong, Diana J. M. van den Wollenberg, Pierre Boulanger, Rob C. Hoeben

**Affiliations:** 1 Department of Molecular Cell Biology, Leiden University Medical Center, Leiden, The Netherlands; 2 Department of Neurosurgery, Brain Tumour Center, Erasmus MC, 3015 CE Rotterdam, The Netherlands; 3 UMR754-INRA-EPHE, Unit of Viral Infections and Comparative Pathology, University of Lyon, Lyon, 69007 France

## Abstract

The mammalian orthoreovirus Type 3 Dearing has great potential as oncolytic agent in cancer therapy. One of the bottlenecks that hampers its antitumour efficacy *in vivo* is the limited tumour-cell infection and intratumoural distribution. This necessitates strategies to improve tumour penetration. In this study we employ the baculovirus *Autographa californica* multiple nucleopolyhedrovirus as a tool to expand the reovirus’ tropism and to improve its spread in three-dimensional tumour-cell spheroids. We generated a recombinant baculovirus expressing the cellular receptor for reovirus, the Junction Adhesion Molecule-A, on its envelope. Combining these Junction Adhesion Molecule-A-expressing baculoviruses with reovirus particles leads to the formation of biviral complexes. Exposure of the reovirus-resistant glioblastoma cell line U-118 MG to the baculovirus-reovirus complexes results in efficient reovirus infection, high reovirus yields, and significant reovirus-induced cytopathic effects. As compared to the reovirus-only incubations, the biviral complexes demonstrated improved penetration and increased cell killing of three-dimensional U-118 MG tumour spheroids. Our data demonstrate that reovirus can be delivered with increased efficiency into two- and three-dimensional tumour-cell cultures via coupling the reovirus particles to baculovirus. The identification of baculovirus’ capacity to penetrate into tumour tissue opens novel opportunities to improve cancer therapy by improved delivery of oncolytic viruses into tumours.

## Introduction

The wild-type mammalian orthoreovirus (RV) type 3 Dearing (T3D) is under investigation as oncolytic agent in pre-clinical research and phase I, II and III clinical trials^1^. The RV species belongs to the genus *Ortheoreovirus* within the family of *Reoviridae*, characterized by a segmented dsRNA genome packaged into a double-layered icosahedral-shaped protein capsid. The canonical cellular receptor for reovirus is Junction Adhesion Molecule-A (JAM-A)^2^. JAM-A is a type I transmembrane protein with two extracellular immunoglobulin domains and a short cytoplasmic tail. The protein is concentrated at the apical region of intercellular tight junctions of epithelial and endothelial cells^3^. After sialic acids-mediated attachment of RV to the host cell, the RV’s spike protein σ1 engages JAM-A to establish a high-affinity interaction.

The mechanism of RV T3D oncolysis can be primarily attributed to its unrestricted replication and killing of tumour cells bearing genomic mutations that lead to active RAS signalling via RalGEF/p38 downstream pathways, while in untransformed, healthy cells the reovirus’ infectious cycle is limited by the dsRNA-activated protein kinase R (PKR)-mediated antiviral responses^4,5^. Additionally, other cellular determinants as receptor availability and virus uncoating efficiency have been shown to influence RV’s ability to kill cancer cells^6–10^.

The reovirus-induced tumour eradication *in vivo* is a result of both the direct cytolytic effect of the virus and indirect tumour killing in response to viral-induced innate and adaptive immune responses. Replication of the oncolytic-virus increases anti-tumour immunity, thereby enhancing the therapeutic efficacy of RV^11,12^.

To date more than 30 clinical trials exploiting RV for tumour treatment are ongoing or have been completed^1^. RV demonstrates an outstanding safety profile and anti-tumour efficacy has been witnessed in several cancer types. In these studies RV is used either as monotherapy or in combination with conventional treatment^13^. Although safe, many patients show partial and transient responses to the treatment, making further improvement of RV-based cancer treatment necessary^11,12^.

Several hurdles that hamper antitumour efficacy have been defined. Systemic delivery can be thwarted by, for instance, circulating antibodies against RV, activation of the innate immune system by pathogen-associated molecular patterns (PAMPS) on the virus, and high interstitial fluid pressure which hampers the extravasation of the virus^14,15^. Even if substantial amounts of virus particles enter the tumour after intratumoural administration, clearance of the entire tumour is still not ensured^12,16^. Physical barriers posed by the stromal compartment, including the extracellular matrix, as well as antiviral immunity may limit the distribution of the virus^14,15^. Moreover, RV’s ability to enter tumour cells may be negatively affected by the scarcity and inaccessibility of its cellular receptor JAM-A, although it remains to be established how important this factor is, taking into account the existence of alternative, e.g. JAM-A-independent, entry mechanisms^17,18^.

In our efforts to identify strategies that can improve RV’s applicability and oncolytic potency, we selected baculovirus (BV) as a potential ally. BVs are insect viruses with a very narrow host range. BVs exhibit in two distinct phenotypes during their natural infection cycle, the occlusion-derived viruses (ODV) that mediate the horizontal transmission between insect hosts and the budded viruses which are produced by the host’s midgut epithelial cells, and establish systemic infection inside the insect. The formation of ODV critically relies on the viral capacity to produce the polyhedrin protein. In biotechnology application, polyhedrin deletion mutants are employed that can only form the rod-shaped, membrane-enveloped budded BVs. These BVs gained their popularity in production platforms for recombinant protein production and as gene-delivery vehicles^19^. BV’s circular double-stranded DNA genome (134kbp) is relatively easy to engineer and can harbour large transgenes. BV can be modified for the efficient expression of heterologous transgenes in a broad panel of mammalian, bird, and fish cells, however the virus is unable to replicate in these species. Considering this inability to replicate in mammals and the fact that it is not pathogenic to humans, BV is regarded as fairly safe to use in human cells^19^, and as a safe replication-defective gene-transfer vector for use in humans^20^. The most commonly used BV is the *Autographa californica* multiple nucleopolyhedrovirus (AcMNPV), isolated from an alfalfa looper in the early 1970s^21^.

It has been shown that the cellular receptor for a large number of Adenovirus (AdV) species, the Coxackievirus and Adenovirus receptor (CAR) can be expressed on the baculovirus AcMNPV envelope, creating BV^CAR^ virions. This enabled AdV particles to bind to the baculovirus AcMNPV envelope, forming BV^CAR^-AdV complexes^22^. Cells that were resistant to HAdV-5 vectors carrying a green fluorescent protein (GFP) reporter (AdV.GFP) turned GFP positive upon administration of the BV^CAR^-AdV.GFP complexes. This demonstrates that the AdV was able to enter cells by piggybacking on the recombinant BV^CAR^ virus. Additionally, in a separate study it was demonstrated that BV, harbouring the EGFP gene under control of the human cytomegalovirus (hCMV) immediate early promoter, was able to penetrate through several cell layers into tumour-cell spheroids and into prostate cancer xenografts in a murine model^23^.

Together, this prompted us to study the concept of enhancing RV’s cell entry and tissue penetration capacity by piggybacking on BV virions. Therefore we expressed RV’s cellular receptor JAM-A onto the BV envelope, allowed RV to bind to the BV^JAM^ viruses, and tested the resulting BV^JAM^-RV complex for reovirus infection of JAM-A-deficient U-118MG glioma cells in two-dimensional standard cell-culture conditions and on three-dimensional tumour-cell spheroid cultures. We show that the BV^JAM^-RV complexes facilitate infection and replication of RV and increase cell killing in U-118 MG glioblastoma cells which are refractory to RV under normal cell culture conditions^17^. Moreover, RV achieved deeper penetration and spread into spheroids, and increased spheroid cell killing upon administration of RV complexed with BV^JAM^.

## Results

### Expression of JAM-A on the baculovirus AcMNPV envelope

**Figure 1:**
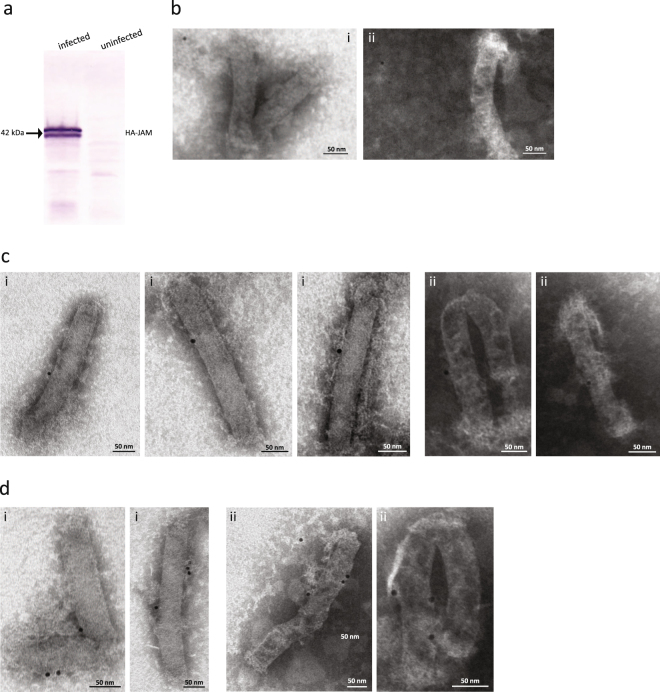
RV’s cellular receptor JAM-A is present on the BV envelope. (**a**) Western blot analysis of Sf9 cells infected with BV^JAM^. The blot was reacted with mAb α-HA followed by antibody-alkaline phosphatase, to detect the presence of JAM-A in infected cells. (**b–d**) Electron microscopic images of BV^JAM^, negatively stained using uranyl acetate and incubated with mouse mAb α-JAM-A (i = sc-53623, ii = ab17261) followed by α-mouse antibody tagged with 10-nm colloidal gold. (**b**) Example of a BV^JAM^ field on grid showing low background labelling. (**c**,**d**) Enlargements of BV^JAM^ virions bearing respectively a single (**c**) or multiple (**d**) gold grains.

To generate a BV vector that carries the RV receptor JAM-A on its envelope, a recombinant BV was engineered that carries the human JAM-A cDNA under control of the BV polyhedrin promoter. The full-length JAM-A gene including a human influenza hemagglutinin (HA) tag was inserted in the pBlueBac4.5/V5-His vector. After generation, production and purification of the recombinant baculovirus BV^JAM^, expression of the JAM-A protein on the BV^JAM^ envelope was confirmed by Western blot analysis using an α-HA antibody on the lysate of BV^JAM^ infected Sf9 cells (Fig. 1a). The double band around 42 kDa is probably due to incomplete N-linked glycosylation of the JAM-A protein at position 185 in Sf9 cells^24^. While baculovirus-insect cells are capable of N-linked glycosylation of proteins, the glycosylation in these cells is incomplete compared to mammalian cells^25^.

Subsequently, we examined the BV^JAM^ virions by immune-electron microscopy (EM). Virions were applied to an EM grid and incubated with α-JAM-A antibodies ab17261 or sc-53623, followed by a 10-nm colloidal gold-tagged secondary antibody and analysed by EM microscopy (Fig. 1b–d). Low background labelling is observed for both antibodies (Fig. 1b). Most BV^JAM^ virions observed held one (Fig. 1c), or more gold grains per virion (Fig. 1d). Although the number of gold grains attached to a BV does not necessarily correlate to the number of JAM-A molecules on the envelope, it can be concluded that JAM-A was efficiently expressed at the surface of BV^JAM^ virions.

### BV^JAM^ formed a complex with RV and facilitated infection of RV in U-118 MG cells

**Figure 2:**
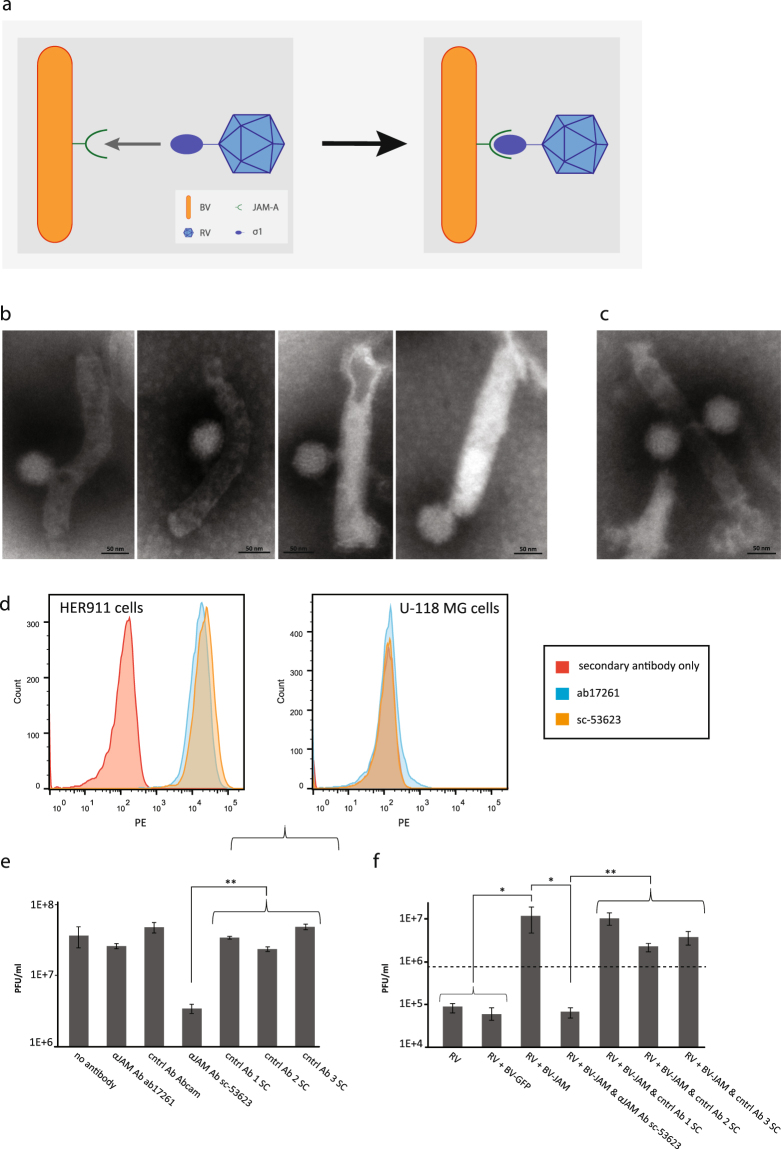
RV and BV^JAM^ associate and form a biviral complex. (**a**) Schematic representation of the BV^JAM^-RV complex. RV attachment protein σ1 bound to JAM-A expressed on the BV envelope. (**b**,**c**) Electron microscopy images of BV^JAM^-RV complexes. The virions were negatively stained with uranyl acetate. Most complexes consisted of one BV^JAM^ and one RV virion (**b**), some complexes showed other combinations of single or multiple BV^JAM^ and RV virions (**c**). (**d**) Flow cytometry analyses of recognition of JAM-A on HER911 cells and U-118MG cells as negative control, by α-JAM-A antibodies ab17261 and sc-53623. (**e**) The RV yields from HER911 cells and culture medium upon incubation with α-JAM-A antibodies ab17261 and sc-53623 or as controls unrelated antibodies of the same provider as controls. The error bars represent the standard deviation (n = 3), p values: ** ≤ 3E-4 (**f**) The RV yields from U-118 MG cells and culture medium after incubation with BV^JAM^ or BV^JAM^ exposed to α-JAM-A antibodies sc-53623 or unrelated control antibodies of the same provider. The dashed line represents RV input. The error bars represent the standard deviation (n = 3), p values: * ≤ 4.4E-2, ** ≤ 8.8E-3.

To investigate whether RV was capable of binding to BV^JAM^ to form a BV^JAM^-RV virus complex, we analysed the biviral complex by EM (Fig. 2a). Mostly binary complexes were observed where one RV was bound to one BV^JAM^ (Fig. 2b) or more RV were found to associate with a BV^JAM^ virion (Fig. 2c). To examine whether the formation of the BV^JAM^-RV complexes was dependent on the presence of JAM-A in the BV capsid we performed an antibody blockage experiment on the JAM-A negative glioblastoma cell line U-118 MG. Under standard cell culture conditions these glioma cells are resistant to RV as infection strictly depends on expression of JAM-A on the cell surface^26^. First, two different α-JAM-A antibodies, ab17261 and sc-53623, were tested for their capacity to recognize JAM-A on the JAM-A-positive cell line HER911 by flow cytometry. JAM-A-negative U-118 MG cells served as negative control. Both ab17261 and sc-53623 α-JAM-A antibodies were able to bind to JAM-A on the surface of HER911 cells, whilst the signal for both antibodies on U-118 MG cells did not exceed background level (Fig. 2d). Subsequently, both antibodies were examined for their ability to inhibit RV infection on RV-permissive HER911 cells. Cells were incubated with α-JAM-A antibodies or with unrelated control, antibodies of the same provider, followed by infection with RV. Two days post-infection, cells and medium were harvested and the RV yield was determined by plaque assays on HER911 cells. The α-JAM-A antibody ab17261 did not impede RV infection in HER911 cells as the virus yield was similar to the control samples that were not exposed to antibodies or pre-incubated with unrelated antibody controls. In contrast, HER911 cells incubated with α-JAM-A antibody sc-53623 prior to RV infection exhibited a ten-fold decrease in RV infectious titer compared to the control samples (Fig. 2e). This demonstrated that infection of HER911 by RV is dependent on the availability of JAM-A on the surface and that the RV infection could be inhibited by blocking the receptor with antibody sc-53623. Hence, the α-JAM-A antibody sc-53623 was used in the confirmatory antibody blockage experiment on U-118 MG cells.

In this experiment BV^JAM^ particles were exposed to α-JAM-A antibody sc-53623, unrelated control antibodies of the same provider or no antibodies prior to incubation with RV. Subsequently, this mixture was administered to U-118MG cells. Cells exposed to RV alone or RV incubated with BV-GFP (lacking JAM-A), without antibody pre-incubation, were used as controls. Two days post-infection, cells and medium were harvested and the infectious RV titer was determined by plaque assays on HER911 cells. While the RV yield on U-118 MG cells in the control conditions did not exceed the input level, the RV yield in cells exposed to RV incubated with BV^JAM^ was 100-fold higher (Fig. 2f). This increase in RV yield was completely abolished upon exposure of U-118 MG cells to RV which was incubated with BV^JAM^ pre-exposed to α-JAM-A antibodies, while pre-incubation of BV^JAM^ with unrelated control antibodies prior to RV addition did not have an effect on the RV yield. Additionally, the RV yield was fully preserved upon addition of α-JAM-A antibodies after incubation of BV^JAM^ with RV (data not shown). These results provided evidence that RV was able to bind to BV^JAM^ and formed a virus complex. These complexes facilitated RV infection in U-118 MG cells that otherwise resist wild-type RV infection.

**Figure 3:**
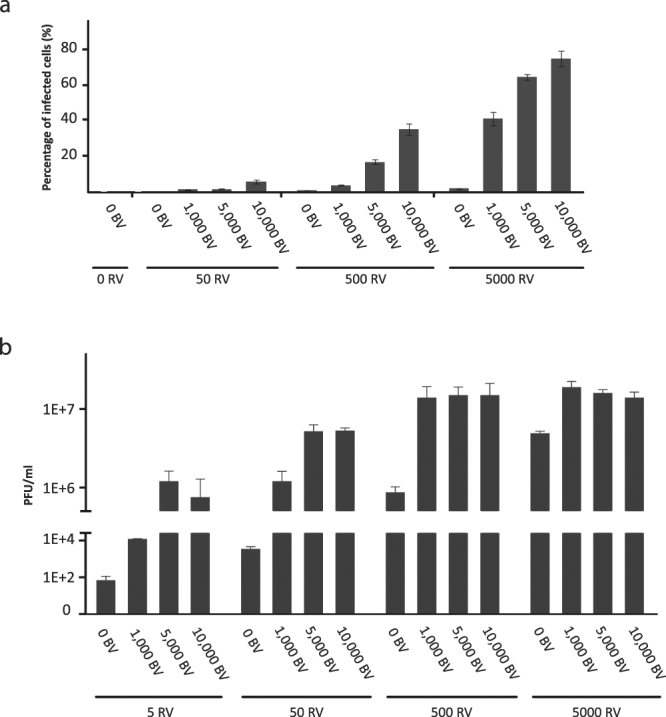
Complexing BV^JAM^ and RV increases RV infection of U118-MG cells. (**a**) Flow cytometry analyses of the fraction of RV infected U-118 MG cells, stained for RV capsid protein σ3, upon incubation with either RV or with different ratios of BV^JAM^-RV complexes 40 h post-infection. The error bars represent the standard deviation (n = 2). (**b**) RV yields from U-118 MG cells upon infection with either RV or with different ratios of BV^JAM^-RV complexes as determined by plaque assays. The error bars represent the standard deviation (n = 3), for BV^JAM^-RV ratios 0:5 and 1,000:5 (n = 2).

To further study the BV^JAM^-mediated RV infection we assessed the fraction of RV-infected U-118 MG cells upon exposure to BV^JAM^-RV by flow cytometry. Various ratios of viral particles (vp) of RV and BV^JAM^ were complexed prior to addition to U-118 MG cells. The cell population was harvested at 40 h post-incubation, fixed and stained for the presence of RV capsid protein σ3. Upon administration of RV alone, 1% of the cells became RV positive at the highest dose of 5000 vp per cell (Fig. 3a). Exposure of U-118MG cells to BV^JAM^-RV in different ratios (50–5,000 vp RV per cell and 0–10,000 vp BV per cell) increased notably the percentage of RV infected cells up to 75%. Presumably, the actual percentage of infected cells was even higher at the highest virus concentrations applied (5,000 vp RV and 10,000 vp BV per cell) as the majority of cells already succumbed to the virus infection and only 2,500 cells could be measured by the flow cytometer (compared to 10,000 cells for the other conditions). Furthermore, it was apparent that the concerted increase of RV and BV^JAM^ particles led to an increase in the percentage of RV-infected cells.

To assess the productive replication of RV in U-118MG cells upon infection with BV^JAM^-RV or with RV alone, the RV infectious yields from the cells were analysed four days post-infection. The addition of BV^JAM^ had the most substantial effect on the RV yield at low concentrations of RV; at 5 and 50 vp RV per cell the addition of BV^JAM^ increased the yield more than 10,000- and 1,000- fold, respectively (Fig. 3b). At higher RV concentrations (500 and 5,000 vp per cell), the yield increase was respectively fifteen- and four-fold upon incubation with BV^JAM^. Notably, at these higher RV concentrations the difference in yield between applying 1,000 or 10,000 BV^JAM^ particles per cell was relatively minor. However, at lower RV concentrations the increase was stronger, presumably because progeny RV virions produced by the U-118 MG cells could engage with residual free BV^JAM^ particles, thereby allowing the progeny viruses to productively infect uninfected cells.

### Cell viability of U-118 MG cells was decreased via apoptosis after RV infection supported by BV^JAM^

**Figure 4:**
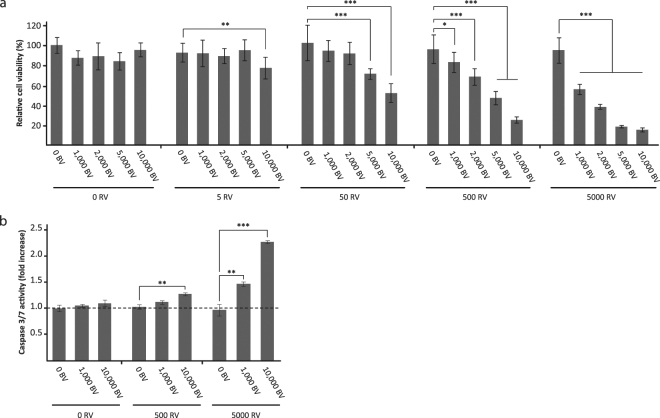
Enhanced RV-mediated killing of U-118 MG cells by apoptosis upon infection with BV^JAM^-RV. (**a**) Analyses of the cell viability of U-118 MG cells by WST-1 assay at four days post-infection with BV^JAM^, RV, or BV^JAM^-RV complexes. Uninfected cells were set at 100% viability. The error bars represent the standard deviation (n = 9), p values: * = 3.9E-2, ** = 4.8E-3, *** < 1E-4. (**b**) Caspase-3 and -7 analysis in the lysate of U-118 MG cells upon infection with BV^JAM^, RV or BV^JAM^-RV complexes. Measurements were normalized to the caspase-3 and -7 values in uninfected cells. The error bars represent the standard deviation (n = 3), p values: ** ≤ 2.1E-3, *** < 1E-4.

The BV^JAM^-RV complex facilitates infection and replication of RV in U-118 MG cells. Subsequently, U-118 MG cells were used to evaluate cytolytic activity of RV upon administration with BV^JAM^ at five days post-infection by a cell viability assay (Fig. 4a). Administration of BV^JAM^ without RV did not significantly affect cells viability, even at the highest concentration of BV^JAM^ (10,000 vp per cell) (p = 0.17). Also the infection of U-118 MG with RV alone, in concentrations ranging from 0 to 5,000 vp per cell, did not reduce cell viability compared to the control cells (p = 0.30 at 5,000 vp per cell). In contrast, exposure of the cells to the BV^JAM^-RV complex caused substantial cell killing in U-118 MG cells. At a BV^JAM^-RV ratio of 10,000 – 5 vp per cell a significant decrease in cell viability compared to the RV only condition could be observed. Both the increase of RV and of BV^JAM^ particles decreased U-118 MG cell viability. As BV is not able to replicate in mammalian cells and is not cytolytic, this decrease in viability can be attributed to RV-mediated cell killing.

Next, we studied whether the BV-mediated RV infection induced cell killing via apoptosis. Therefore the enzymatic activity of two key effectors in the apoptotic pathway, caspase-3 and -7, was measured in the lysate of U-118 MG cells at four days post-infection. Administration of BV^JAM^ or RV only did not lead to induction of apoptosis in the cells (Fig. 4b). However, incubation of cells with biviral BV^JAM^-RV increased activity of caspase-3/7, demonstrating an advanced stage of apoptosis in the infected cells. Taken together, our data showed improved RV-mediated infection and cell killing of two-dimensional U118MG cultures after co-incubation of RV with BV^JAM^.

### Enhanced RV penetration and cell killing of U-118 MG spheroids upon administration in complex with BV^JAM^

To explore whether the BV^JAM^-RV complexes can stimulate RV transduction in three-dimensional spheroid cultures of U118 MG cells, the penetration depths of RV into spheroids, upon administration with BV^JAM^, were evaluated as a surrogate for the RV infection and penetration potency.

**Figure 5:**
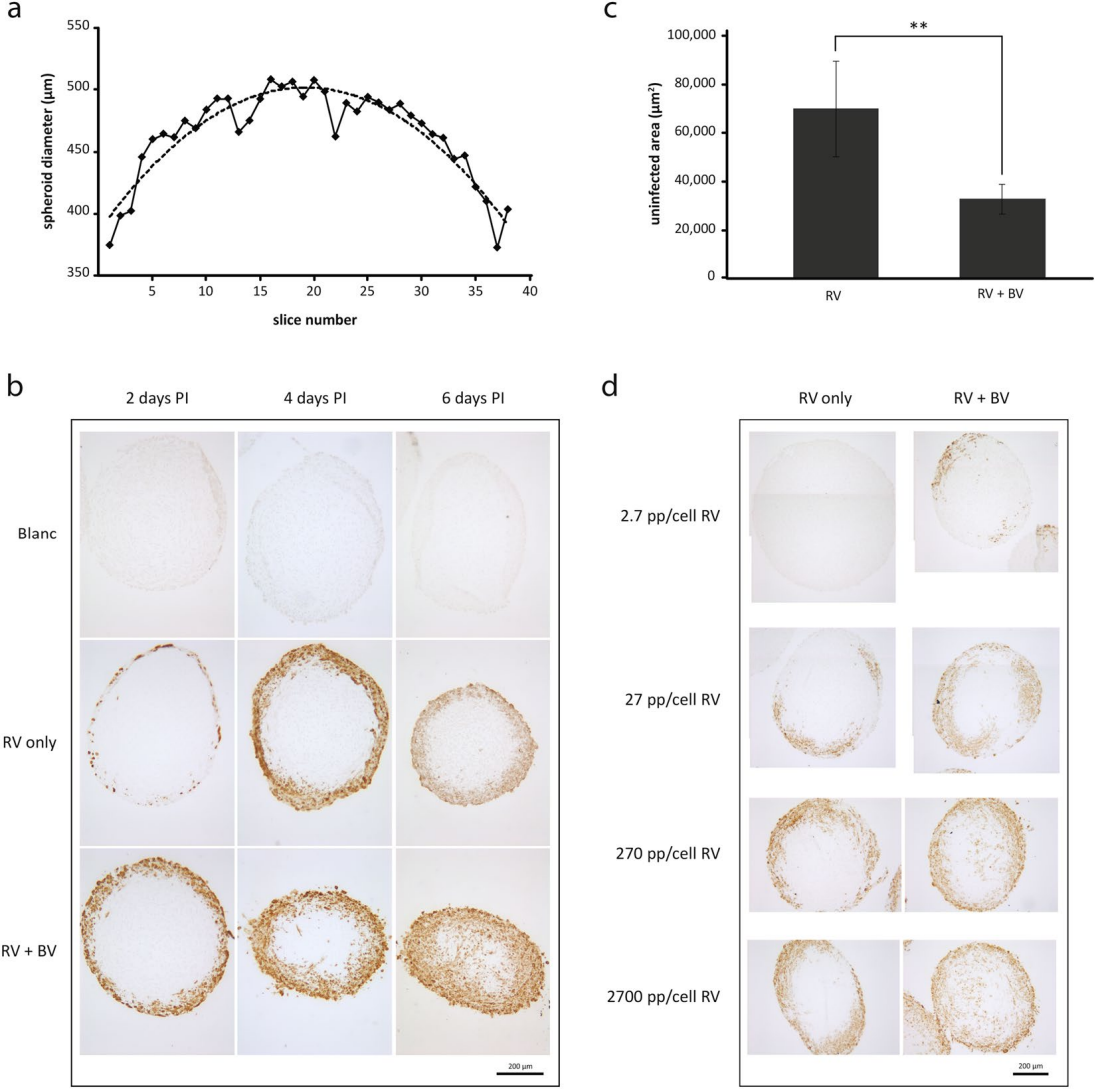
Improved penetration and spread of RV in U-118 MG spheroids upon infection with BV^JAM^-RV. (**a**) Example of a graph assigning the midpoint of a spheroid. After the spheroids (25,000 cells at start) were fixed and sectioned, the diameter of each of the spheroid sections was measured. These values were combined in a graph as represented here and the trend line through the data points was used to determine the spheroid section with the largest diameter. (**b**) Representative images of RV penetration analysis in U-118 MG spheroids, either as single agent (10,000 vp/cell) or in combination with BV^JAM^ in a RV:BV^JAM^ ratio of 1:2.5. Two, four and six days post-infection spheroids were fixed, sliced and stained for the presence of RV capsid protein σ3 by immunocytochemistry. (**c**) Uninfected area of spheroids infected as in (b) with RV only or the BV^JAM^-RV complex at six days post-infection. For each spheroid the uninfected area of three to five middle sections was measured and averaged. The error bars represent the standard deviation (n = 4 spheroids), p value: ** = 9.2E-3. (**d**) Representative images of RV titration experiment on spheroids infected with RV only or in the presence of a surplus of BV^JAM^ at six days post-infection through immunocytochemistry staining for RV capsid protein σ3.

To compare the penetration depths of the vectors, it is essential to identify the spheroid’s midpoints. Hence, the spheroids were fixed after harvesting, sliced into sections and the diameter of each section was measured. These values were graphically plotted and a trend line was set to pinpoint the section with the largest diameter, representing the middle section of the spheroid. See Fig. 5a for an illustration of this procedure.

To evaluate the penetration depth of RV alone (10,000 vp per cell) or in combination with BV^JAM^ (ratio RV:BV^JAM^ = 1:2.5) over time, the spheroids (25,000 cells at start) were harvested at two, four and six days post-infection. In the middle sections the extent of reovirus infection was visualized by staining for the presence of RV capsid protein σ3. As described previously, RV as a single agent is able to infect spheroids composed of U-118 MG cells whilst U-118 MG monolayer cultures are resistant to infection with RV. This effect was attributed to factors that are secreted by the spheroids, such as cathepsin B and L^17^. It is therefore not surprising to observe that RV as single agent caused infection in U-118 MG spheroids (Fig. 5b). However, when BV^JAM^-RV complexes were applied, this resulted in an increased infection of cells in the outer rim of the spheroids, observed just after two days. Moreover, there were RV infected cells deeper in the spheroid showing that RV had penetrated deeper into the spheroid in combination with BV^JAM^. This effect was even more pronounced at four and six days post-infection. In spheroids incubated with BV^JAM^-RV the RV-infected cells were present throughout the entire spheroid at six days post-infection and reached the centre of the spheroid, whereas incubation with RV only led to infection of the outer rim. Note that the spheroids exposed to RV and BV^JAM^-RV became smaller over time as compared to uninfected spheroids, which was caused by detachment of dead cells from the spheroids. Quantification of the difference in penetration of RV administered alone or in complex with BV^JAM^ was accomplished by measuring the uninfected (unstained) centre area of the spheroid. From Fig. 5c it becomes apparent that at six days post-infection the uninfected area of spheroids slices infected with RV alone was twice the size of the uninfected area of spheroids slices infected with the BV^JAM^-RV complex. In a subsequent experiment, the effect of increasing RV amounts (2.7–2,700 vp per cell) with a surplus of BV^JAM^ particles (30,000 vp per cell) onto spheroids was evaluated at six days post-infection. Although infection with RV alone could not be detected at 2.7 vp per cell, the spheroids stained positive for RV from 27 vp per cell onwards (Fig. 5d). However, the virus could only be detected in the outer rim of the spheroid and did not reach the spheroid centre, even at the highest concentration of 2,700 vp per cell. If administered with a surplus of BV^JAM^ particles, the spheroids showed patches σ3 positive cells evidencing RV infection already at 2.7 vp per cell. With increasing RV concentrations, infections extended deeper into the spheroid and at 270 and 2,700 RV vp per cell σ3 staining was evident in the centre of the spheroids. Together, these data showed that BV^JAM^ facilitated RV penetration and spread throughout the spheroids.

**Figure 6:**
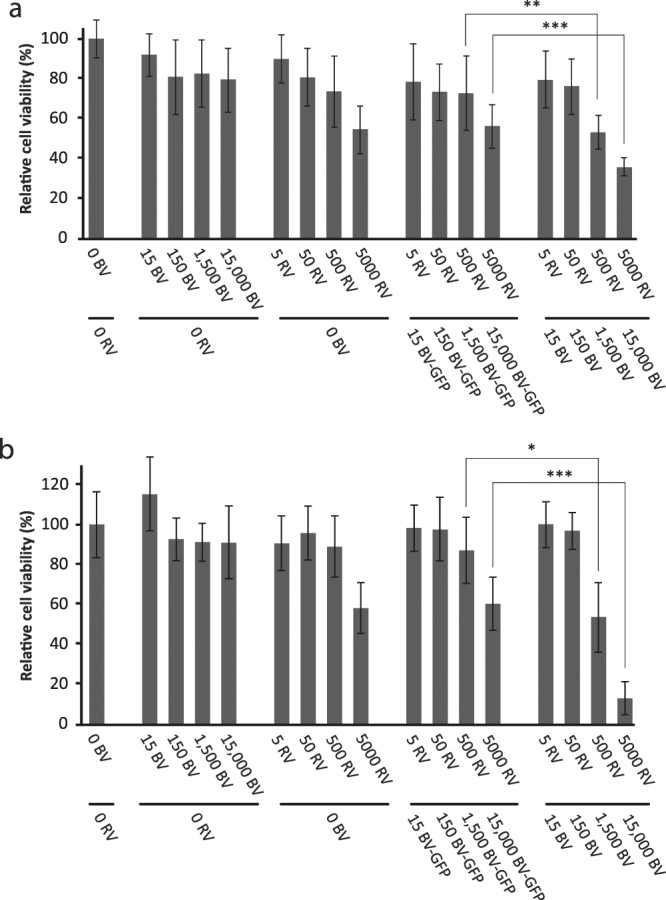
Improved cell killing of U-118 MG spheroids upon infection with BV^JAM^-RV. Spheroids of 25,000 (**a**) or 5,000 (**b**) cells at start were either mock treated or transduced with BV^JAM^ only, RV only or RV pre-incubated with BV^JAM^ or BV-GFP. At six days post-infection the viability of the spheroids was assessed by WST-1 assay. Mock-treated cells were set at 100% viability. The error bars represent the standard deviation (n = 8), p values: * = 3.1E-2, ** = 1.6E-2, *** = 2E-4.

Furthermore, we assessed whether administration of the BV^JAM^-RV complex led to increased cell death in spheroid cultures compared to RV used as single agent. To this end, the viability of spheroids of 25,000 cells (25 K) and 5,000 cells (5 K) was analysed at six days post-infection. Incubation of spheroids with RV only, BV^JAM^ only or RV combined with BV-GFP served as controls. Both 25 K and 5 K spheroids that were exposed to BV^JAM^ showed a slight decrease in viability (Fig. 6a,b). However, this effect did not correlate to the BV^JAM^ dose applied. The highest concentration of RV (5,000 vp per cell) reduced the viability of the spheroids of both sizes considerably (to around 60%). A similar reduction in viability was obtained upon exposure of spheroids to RV plus BV-GFP. As these viruses were not able to form a complex, this showed that solely the presence of BV particles in a sample with RV was not sufficient to cause an additional decrease in viability of the spheroids. In contrast, incubation of the spheroids with the BV^JAM^-RV complex resulted in a significant decrease in cell viability compared to application of RV only. This decrease was most pronounced in spheroids of 5,000 cells. At ratio 500 RV–1,500 BV^JAM^ vp per per cell we observed a 20% increase in cell killing as compared with the RV only condition for the 25 K spheroids and a 33% increase in the 5 K spheroids and for 5000 RV–15,000 BV^JAM^ vp per cell this increase in cell death was respectively 22% versus 47%. Together these results demonstrate that RV in complex with BV^JAM^ penetrated deeper into the spheroids and caused more tumour cell death than RV only under these conditions.

## Discussion

In a previous study, Granio *et al*. elegantly provided proof-of-concept of binding adenovirus particles on CAR-exposing BV, to increase the transduction of cell lines that are normally refractory to AdV infection^22^. Here we show for the first time that RV complexed to its receptor JAM-A expressed on the BV envelope transduces and kills the RV-resistant U-118 MG glioma. In spheroid cultures BV^JAM^ facilitated the RV to penetrate deeper into the three-dimensional U-118 MG cell mass, causing increased cell killing.

An effective oncolytic virotherapy must overcome the various hurdles that hamper therapeutic efficacy. A strong initial infection and rapid spread of the therapeutic virus can enhance anticancer efficacy^15,27^. As became apparent from our studies in three-dimensional tumour spheroids, BV assisted RV in two interrelated aspects; (i) more cells became RV positive, already observed early after infection and (ii) RV penetrated deeper into the spheroids.

The increased cell killing together with improved spreading/penetration could potentially contribute to more effective immunogenic lysis of the infected cells buried inside a tumour, allowing more and additional tumour-associated antigens to be released. These neoantigens could enhance tumour-antigen processing and presentation by professional antigen presenting cells, eventually culminating in a more potent immune response against the tumour cells^12,28^.

BV^JAM^ viruses were produced in Sf9 cells after infection with recombinant BV expressing JAM-A under the control of the polyhedrin promoter. We demonstrated expression of the JAM-A protein on the BV envelope by EM analysis. The majority of JAM-A positive BVs showed only few immunogold grains per virion. Also, predominantly single RVs were shown bound to BV^JAM^ virions. These findings mimic the EM data shown by Granio *et al*. in their study on BV^CAR^-AdV complexes. These authors compared expression of CAR- and BV membrane protein GP64, which showed similar numbers of gold grains and, similarly to our results, mostly single CAR-staining gold particles per virion. As GP64 is an intrinsic BV protein, this illustrated that the immunogold labelling of the acquired CAR protein was equally potent. Although RV and AdV originate from distinct virus families, with a different genetic background and limited similarities in their infectious mechanism, both viruses show a similar mechanism of attachment to host cells. Both JAM and CAR are glycoproteins belonging to the immunoglobulin superfamily and form homodimers with structural similarities which are expressed at regions of cell-cell contact^29^. Moreover, the trimeric attachment proteins of RV and AdV, σ-1 and fiber, have comparable structural regions and are able to interact with their receptors in a similar manner through high affinity binding (dissociation constant, K_D_, of 0.9–2.4 nM for RV^2,30^ and 2–6 nM for AdV^31,32^)^33,34^. It is therefore conceivable that the JAM-A expression on the BV envelope and the binding kinetics of BV^JAM^-RV reflected the situation of CAR expression of BV and the binding of AdV to BV^CAR^.

The mechanism of BV entry into mammalian cells has been thoroughly studied and is mediated by the BV GP64 major envelope fusion protein. This trimeric glycoprotein, which is found polarized on the end of the BV where both budding and cell entrance initiates, is responsible for the attachment to the mammalian host cell and the subsequent process of virus internalization^35,36^. In this study we showed that, in contrast to RV only, the BV^JAM^-RV complex was able to establish a productive RV infection in U-118 MG cells, indicating that the capacity of the BV GP64 protein to attach to and enter the U-118 MG cell was not obstructed by the RV cargo.

It remains to be clarified how BV^JAM^-RV complexes internalize into the JAM-A deficient U-118 MG cells. Separately, both RV and BV are thought to enter mammalian cells via clathrin-mediated endocytosis^37,38^, although for both viruses also other endocytic pathways are suggested in the cellular uptake process^18,35^. Considering that the size of the BV^JAM^-RV complex exceeds the 200 nm size limit for the diameter of clathrin-dependent endocytic vesicles, it could not be excluded that the internalization of biviral complexes occurred via macropinocytosis- or phagocytosis-mediated endocytosis. An EM study using cell transduction by baculovirus-human adenovirus complexes (BV^CAR^-AdV^22^) has shown that the attachment of BV to the cell surface induces the formation of clathrin-coated vesicles. However, at later times of the viral entry process, EM analysis also showed BV^CAR^ and HAdV5 particles in large intracytoplasmic vesicles of which size and morphology corresponds to macropinocytic or phagocytic vesicles^22^.

Together, these results suggested that, similar to the BV^CAR^-AdV complexes, the cell internalization of the BV^JAM^-RV complex can result from two co-existing mechanisms, clathrin-dependent and clathrin-independent endocytosis. This hypothesis is supported by many observations that a given virus can infect cells via different endocytic pathways, as described above for RV and BV^18,33^ as is, for example, shown for human adenovirus which can use both clathrin-mediated uptake and macropinocytosis^39–41^. Inside the endocytic compartment, RV is subject to conformational transformations, characterized by the loss of several capsid proteins including attachment protein σ1^42^. At this stage in the infection cycle we expected RV to lose the connection with BV^JAM^ and migrate to the cytoplasm alone. This is in agreement with the results obtained by Granio *et al*., illustrating by EM that the BV^CAR^-AdV complex is internalized as a whole and did not separate until they were in a cytoplasmic vesicle from which AdV escaped separate from BV^CAR^ ^22^.

Although investigating the mechanism by which BV is able to penetrate deep into tumour spheroids is beyond the scope of this study, we scrutinized the literature for potential clues.

In the natural infection cycle of BV AcMNPVs, progeny BV viruses escape the midgut epithelial cells on the basal side. From there, two main routes of systemic virus spread have been proposed: (i) the virus directly penetrates the basal lamina secreted by midgut cells to reach the underlying tracheal branches (the respiratory system of the insect). (ii) the tracheal cells reach into the basal lamina of the midgut cells and the virus would need to penetrate the basal laminae secreted by the tracheal cells to establish systemic spread^43–45^. In either theory, the virus would need to cross a basal lamina. Basal laminae or basement membranes are a specialized type of extracellular matrix (ECM) present in both invertebrates and vertebrates and consist of flexible sheets, build up from different proteins (e.g. collagen type IV, laminin and fibronectin), that form a more compact and dense matrix network than other types of ECM^46,47^.

Although the precise process by which BV penetrates the basal lamina of the tracheal cells is not fully elucidated, several studies point to a crucial role for the BV-encoded viral fibroblast growth factor (vFGF). BVs are the only viruses that produce FGF. The vFGF is made early in BV-infected insect cells and is shuttled to the cell surface where it anchors to heparin sulphate proteoglycans. Upon budding from the cell, the virus attaches vFGF to the cell-membrane derived envelope^48,49^. It has been shown that vFGF accelerates virus spread and diminishes the time required to kill the insect by initiating a cascade of events (involving matrix metalloproteases and effector caspases) leading to remodelling of the basal lamina, thereby allowing BV to penetrate this layer and infect the underlying tracheal cells^43,48,49^.

In oncolytic virotherapy, the ECM in and around the tumour mass can hamper the spread of the viruses^15^ and we already showed the abundance of ECM in U-118 MG spheroids in a previous study^17^. Therefore we envision a function for the membrane-anchored vFGF in assisting BV to penetrate the ECM surrounding U-118 MG tumour cells and RV, being coupled to BV^JAM^, might take advantage of this mechanism.

The findings that BV is able to spread through tissue and can deliver cargo with therapeutic value are very promising. On the one hand, it would be worthwhile to investigate the mechanism controlling the spreading capacity of BV in a human tumour environment as this instrument may be translated to other oncolytic virus- and gene therapy systems. On the other hand, BV vectors themselves hold great potential for use as gene therapy vectors, not exclusively as a result of their capacity to transduce cells and penetrate tissues, as described in this study and by others^23,50^, but also because it is relatively easy to genetically engineer BVs, for instance to incorporate therapeutic transgenes. The BV-insect cell expression system is already used extensively to produce products for clinical use and considerable pre-clinical efforts are being made on the use of BV for gene therapy purposes^19,27,50–52^. Although still in its infancy, it is plausible that BV vectors will find their way towards patient application^53^. Our findings could be of great significance for such developments.

Taken together, in this study we showed that RV in the BV^JAM^-RV complexes is able to transduce and kill the RV-resistant U-118 MG glioma cells in standard cell culture conditions and showed an enhanced penetration and cell killing capacity in U-118 MG tumour cell spheroid cultures. These results are auspicious, both conceptually, as they demonstrate that there are mechanisms to boost the intratumoural spread of oncolytic viruses, and for their therapeutic implications, as BV’s benefits as a gene therapy vector may be implemented clinically in the future.

## Material and Methods

### Cell lines

The human cell lines U-118 MG (glioblastoma) and HER911 (HAdV-C5 early region 1 transformed human embryonic retinoblasts)^54^ were maintained at 37 °C in a humidified atmosphere of 5% CO_2_, in high-glucose Dulbecco’s modified Eagle’s medium (DMEM, Gibco | Thermo Fisher Scientific, Waltham, MA, USA) supplemented with 8% foetal bovine serum (FBS) (Gibco | Thermo Fisher Scientific) and penicillin-streptomycin (pen-strep) (Gibco | Thermo Fisher Scientific).

The insect cell line *Spodoptera frugiperda* Sf9 was cultured as monolayer in Sf-900 II Serum Free Medium (Gibco | Thermo Fisher Scientific), supplemented with 5% FBS and pen-strep. Routinely, cells were passaged in two-third of new medium and one-third of conditioned medium and placed at 27 °C in a humidified atmosphere without addition of CO_2_.

### Virus production and purification

#### Reovirus

The wild-type orthoreovirus T3D strain R124 (here referred to as reovirus (RV)) was isolated earlier from a stock of reovirus T3D by two rounds of plaque purification using HER911 cells^26^. RV propagation was performed as described before^17^. In brief, HER911 cells were infected with a multiplicity of infection (MOI) of 1 to 3 plaque forming units (PFU^911^) per cell and the medium was replaced at three h post-infection. Cells and medium were harvested at 72 h post-infection and separated by centrifugation (10 min at 3000 g). The cell fraction was resuspended in a small volume of medium and subjected to three cycles of freezing and thawing. After centrifugation, the supernatant was mixed with the medium fraction and subsequently purified by a double discontinuous caesium chloride (CsCl) gradient centrifugation. The virus was isolated from the gradient with a syringe and desalted in an Amicon Ultra-15 Centrifugal Filter Device (molecular weight limit of 100 kDa, Millipore, Billerica, MA, USA). The virus was stored in RV storage buffer (10 mM Tris. HCl pH = 7.5, 150 mM NaCl, 10 mM MgCl_2_·6 H_2_O, 5% sucrose) at −80 °C. The infectious titer of the virus in was determined by plaque assay on HER911 cells and the number of genome copies was determined by measuring the optical density of the sample at 260 nm on the Nanodrop ND-1000 spectrophotometer with conversion factor 2.1E12. The ratio between the RV particles per ml (vp per ml) and the infectious RV virions in PFU per ml ranged from 150:1 to 600:1.

#### Baculovirus

The recombinant baculovirus AcMNPV expressing JAM-A (BV^JAM^) was constructed by incorporation of the human JAM-A gene in the pBlueBac4.5/V5-His vector (Thermo Fisher Scientific, Waltham, MA, USA) under control of the polyhedrin promoter. First, the JAM-A cDNA was isolated from the pcDNA-HA-JAM plasmid (described before in^26^) by overlap extension-polymerase chain reaction (PCR) with oligonucleotides FW-haJAM-NheI (GTTGCTAGCCACCATGGGGACAAAGGCGCAAGTC) and RV-haJAM-AgeI (GTTACCGGTTACACCAGGAATGACGAGGTCTGTTTGA) and ligated into the pCR-Blunt II-Topo vector (Thermo Fisher Scientific) resulting in vector pCR-BluntII-Topo-HA-JAM-A. Plasmids pBlueBac4.5/V5-His and pCR-BluntII-Topo-HA-JAM-A were treated with restriction enzymes *Bsh*TI and *Nhe*I and ligated. This resulted in pBlueBac-HA-JAM-A. Sf9 insect cells were co-transfected with the linearized AcMNPV genome of the Bac-N-Blue™ Transfection Kit and the pBlueBac-HA-JAM-A transfer vector according to the manufacturer’s protocol. The resulting baculoviruses were plaque purified on Sf9 cells and the blue plaques of the recombinant baculovirus BV^JAM^ were detected by X-gal staining. Expression of the JAM-A protein on the selected BV^JAM^ clone was examined by western blot analysis on the lysate of BV^JAM^ infected Sf9 cells using a monoclonal anti-HA antibody (Clone HA-7, H9658, Sigma Aldrich, dilution 1:10,000).

The recombinant baculovirus expressing GFP under the CMV promoter (BV-GFP) was kindly provided by Prof. Monique van Oers (Wageningen University, Wageningen, The Netherlands). BV^JAM^ and BV-GFP propagation was performed by infection of Sf9 cells at a MOI of 1 to 3 with a low-passage seed stock. Supernatants were harvested 72 h post-infection, cleared from cell debris by centrifugation at 900 × g and 4 °C for 10 min and further cleared by ultracentrifugation through a 20% sucrose cushion at 134,000 × g for 1 h at 4 °C. The pellet containing the virus was resuspended in sterile phosphate-buffered saline (PBS) by gentle shaking overnight at 4 °C, before being further purified by ultracentrifugation in a pseudo-linear sucrose-D_2_O gradient. The gradients were generated from a 50% sucrose solution made in D_2_O buffered to pH = 7.2 (solution 1) with NaOH and a 30% sucrose solution made in 10 mM Tris-HCl pH = 7.2, 150 mM NaCl, 5.7 mM Na_2_-EDTA (solution 2). The gradients (10 ml total volume) created by gentle stacking of layers, starting with 1 ml of solution 1 at the bottom, followed by 1 ml of a mixture of 90% solution 1 and 10% solution 2, followed by 1 ml of a mixture of 80% solution 1 and 20% solution 2 etc., ending with 1 ml of solution 2 as last fraction. The virus suspension (maximum of 300 µl) was applied on top and gradients were centrifuged for 18 h at 141,000 × g in a Beckman SW28 rotor. Fractions of 0.5 ml were collected from the top and the infectious BV titer of each fraction was determined by plaque assay on Sf9 cells. The four to five -fractions that contained the highest concentration of infectious BV were pooled and centrifuged at 141,000 × g for 1 h at 4 °C. Supernatant was discarded and the pellet was resuspended in PBS by gentle shaking overnight at 4 °C. The infectious titer was determined by plaque assay on Sf9 cells and the number of genome copies was determined by measuring the optical density of the sample at 260 nm on the Nanodrop ND-1000 spectrophotometer. The BV vp to PFU ratio per ml ranged from 100:1 to 300:1.

### Establishing the BV^JAM^-RV complex

For the generation of BV-RV complexes the virus amounts were based on the viral particle titers. In the experiments, RV was diluted to the desired concentration in culture medium and BV in PBS. The viruses were mixed in a small volume (maximally 50 µl) and incubated at room temperature (RT) for 15–30 min before dilution in the appropriate amount of culture medium and addition to cells in standard cell culture conditions or in spheroid cultures.

### Electron microscopy analyses

Virions of BV^JAM^ were subjected to electron microscopy as described before^22^. Briefly, virions of BV^JAM^ were diluted in 20 µl 0.14 M NaCl, 0.05 M Tris-HCl buffer pH = 8.2 (TBS) before absorbance onto carbon-coated Formvar membranes on electron microscopy grids. Primary antibody monoclonal α-JAM (100 μg per ml, ab17261, Abcam, Cambridge, UK or 200 μg per ml and sc-53623, Santa Cruz Biotechnology Inc., Dallas, Texas, USA) was applied onto the grids and incubated for 1 h. The grids were rinsed with TBS and post-incubated with 10-nm colloidal gold-tagged goat α-mouse immunoglobulin G (IgG) antibody (British Biocell International Ltd., Cardiff, United Kingdom; diluted to 1:50 in TBS) for 30 min at RT. After the specimens were being rinsed with TBS, they were negatively stained with 1% uranyl acetate in H_2_O for 1 min at RT, rinsed again with TBS, and examined under a TEM 1400 JEOL electron microscope (EM) equipped with an Orius-Gatan digitalized camera (Gatan, Grandchamp, France).

The BV^JAM^-RV complexes in a 25:1 ratio were allowed to form as described above, diluted in TBS buffer and applied to the grids prior to the negative staining with uranyl acetate.

### Antibody blockage experiments

To examine the capability of two different α-JAM-A antibodies (100 μg per ml, ab17261, Abcam and 200 μg per ml, sc-53623, Santa Cruz Biotechnology Inc.) to recognize JAM-A on the cell surface, HER911 and U-118 MG cells were dissociated using trypsin (Gibco | Thermo Fisher Scientific), fixed in 4% formaldehyde for 15 min at RT and washed in FACS buffer (0.5% BSA, 2 mM EDTA, 0.09% w/v sodium azide in PBS). Cells were incubated with the primary α-JAM-A antibodies ab17261 or sc-53623 1:400 diluted in FACS buffer for 1 h at RT. Next, cells were washed and exposed to the PE fluorochrome-conjugated goat α-mouse antibody (12-4010-87, F(ab’)2 IgG, Thermo Fisher Scientific) for 30 min at RT in the dark, 1:3000 diluted in FACS buffer. After extensive washing, the cells were resuspended in FACS buffer and assayed on a BD LSRII flow cytometer. Per sample 10,000 events were measured. In the subsequent experiment to determine the ability of both α-JAM-A antibodies ab17261 or sc-53623 to (partly) block a RV infection, HER911 cells in 96-well plates were incubated with 15 μl α-JAM-A antibodies ab17261 or sc-53623 or unrelated antibodies of the same providers (α-fiber, 4D2, Abcam and α-ERAP1 (sc-100727), α-IRF-3 (sc-9082), α-c-Myc (sc-40) from Santa Cruz Biotechnology Inc.) for three h followed by the addition of RV (MOI 3 PFU per cell). Culture medium was replaced at one h post-infection and two days post-infection cells and medium were harvested, subjected to three cycles of freezing and thawing before the infectious RV titer was determined by plaque assays on HER911 cells.

In the confirmatory experiment the U-118 MG cells in a 24-well plate were counted before the start of the experiment. The BV^JAM^ (5000 vp per cell BV^JAM^ in PBS/2%FBS in 20 μl) was incubated for 10 min with 25 μl α-JAM-A antibody sc-53623, Santa Cruz Biotechnology Inc.), three unrelated antibodies (α-ERAP1 (sc-100727), α-IRF-3 (sc-9082), α-c-Myc (sc-40), Santa Cruz Biotechnology Inc.), or as negative control, 25 μl PBS/2%FBS. Next, 2000 vp per cell RV, with a total volume of 20 μl, was added and incubated for 15 min before addition of 475 μl cell culture medium. RV only and RV incubated with BV-GFP virus were used as controls. 500 μl of this solution was incubated with U-118MG for 24 h followed by a replacement of the culture medium. At 48 h post-infection the cells and medium were harvested, three cycles of freezing and thawing were performed and the RV titer was determined by plaque assays on HER911 cells.

### Flow cytometry analyses of reovirus infected cells

U-118 MG cells, in a 24-well plate, were incubated with the BV^JAM^-RV complex or RV alone for 40 h. Next, cells were dissociated with TrypLe Select (Gibco | Thermo Fisher Scientific), fixed in 4% formaldehyde for 15 min at RT and washed in staining buffer (1% FBS, 0.09% w/v sodium azide in PBS, pH = 7.5, filtered). Cells were subsequently permeabilized in Perm/Wash Buffer (BD Biosciences, San Jose, CA, USA) for 15 min at RT and incubated with the primary antibody against RV σ3 protein, 1:200 diluted in Perm/Wash Buffer for 1 h at RT. Cells were washed and exposed to the PE fluorochrome-conjugated rat α-mouse antibody (IgG2a + b, BD Biosciences) for 30 min at RT in the dark, 1:100 diluted in Perm/Wash buffer. After extensive washing, the cells were resuspended in FACS buffer (0.5% BSA, 2 mM EDTA in PBS) and assayed on a BD LSRII flow cytometer. Per sample 10,000 events were measured, only for the highest virus concentrations (5000 vp per cell RV and 10,000 vp per cell BV^JAM^) 2,500 cells per sample were measured as the majority of the cells already succumbed to the virus infection (see Results section). Data were analysed with FACSDiva software (BD Biosciences).

### Determination of reovirus yield

U-118 MG cells were counted and infected with the BV^JAM^-RV complex or RV alone in a 96-well plate. The inoculum was removed by replacement of the culture medium after 24 h. Culture medium and cells were harvested together 4 days post-infection, subjected to three cycles of freezing and thawing and cleared from major cell debris by centrifugation for 10 min at 3000 × g. The RV titer of the supernatant was determined by plaque assays on HER911 cells.

### Caspase assay

Caspase-3 and -7 activity in monolayer U-118 MG cultures upon virus infection was measured by Caspase-Glo® 3/7 Assay (Promega, Winsconsin, USA). Cells were infected with the BV^JAM^-RV complex or RV alone. The inoculum was removed and replaced by fresh culture medium after 24 h. Culture medium was changed again for fresh medium 4 days post-infection before the caspase-3 and -7 activity was assayed according to the manufacturer’s protocol.

### Preparation and infection of spheroid cultures

The establishment of spheroid cultures of U-118 MG cells was described previously in^17^. In brief, the cells were collected from semi-confluent monolayers by trypsin treatment, counted and resuspended in medium containing 2.4 mg per ml methylcellulose (Sigma Aldrich, Saint Louis, Missouri, USA) in the desired concentration (250 or 50 cells per μl). The suspension was added to a U-bottom 96-well plate, 100 μl per well, and the cultures (25,000 or 5,000 cells) were incubated at 37 °C overnight to allow the formation of a spheroid in each of the wells. Methylcellulose was removed by repeated washing with medium and spheroids were incubated with the BV^JAM^-RV complex or RV alone directly after washing.

### Cell viability analyses

Cell viability of monolayer cells or spheroids was assessed at respectively 5 and 6 days post-infection by replacing the culture medium by fresh medium containing 10% WST-1 reagent (Roche, Penzburg, Germany). The cells were re-inserted in the incubator for 1 h (for monolayer cultures) or overnight (for spheroid cultures) before the absorbance was measured in a microplate reader (Bio-Rad model 550, Bio-Rad, Hercules, CA, USA) at a wavelength of 450 nm. The viability measurements were normalized to the viability of uninfected cells.

### Immunocytochemistry analyses of spheroid cultures

Immunocytochemistry analysis of spheroids has been described previously^17^. Shortly, spheroid cultures were fixed overnight in 4% formaldehyde at 4 °C, dehydrated and stained with 1% eosin in ethanol for 10 min. The excess of eosin was removed by washing with 100% ethanol and the spheroids were incubated in xylene two times 15 min before they were embedded in paraffin. Slices of 6 μm were sectioned from the paraffin blocks on a microtome (Leica RM 2165, Nussloch, Germany), transferred to Superfrost Plus slides (Thermo Fisher Scientific) and allowed to dry. The sections were deparaffinised, rehydrated and antigens were retrieved by heating the slides in 10 mM sodium citrate buffer (0.19 g citric acid monohydrate and 1.2 g tri-sodium citrate dihydrate in 500 ml H_2_O) by maintaining the temperature just below the boiling point for 6 min. After cooling, slides were washed twice in water, incubated in 0.3% H_2_O_2_ for 10 min and briefly rinsed in water and PBS afterwards. Samples were exposed to a blocking solution (10% goat serum in PBS) for 1 h at RT before incubation with an antibody 4F2 directed against the reovirus σ3 protein diluted 1:200 in blocking solution for 3 h at RT (monoclonal antibody 4F2 was obtained from the Developmental Studies Hybridoma Bank, maintained by The University of Iowa, Department of Biology, Iowa City, IA 52242). Next, slides were rinsed in PBS and incubated with the polyclonal goat α-mouse antibody conjugated with horseradish peroxidase (HRP) (P0447, Dako, Glostrup, Denmark), 1:50 in blocking solution, for 30 min at RT. The slides were washed thoroughly and the cells were stained with a filtered 3,3-diaminobenzidine (DAB) solution (10 mg DAB dissolved in 10 ml PBS and mixed with 10 ml H_2_O and 10 μl 30% H_2_O_2_) under the microscope. Slides were immersed in water after appearance of the brown colour. After dehydration, the samples were briefly immersed in xylene and mounted in Pertex (Pertex Mounting Medium, Leica Biosystems, Wetzlar, Germany).

### Measuring diameter and un-infected area of spheroids

Measurements on the sectioned spheroids were performed using an Olympus CK40 microscope (Olympus Corporation, Shinjuku, Tokyo, Japan), an Olympus Camedia Digital Camera C-3030 and Olympus software; Olympus DP-soft. To determine the middle of each spheroid, the diameter of the spheroid sections on each slide was measured in two directions, 90 degrees opposite, and averaged. This value was incorporated into a chart and by addition of a trend line the section with the largest diameter, representing the middle of the spheroid, became apparent. The non-infected area was measured from 3-5 middle sections of the same spheroids at six days post-infection and averaged.

### Statistical analysis

The statistical significance of key data was assessed with GraphPad Prism V7.02. The Shapiro-Wilk normality test was used to confirm that the data follow a Gaussian distribution. Thereafter a T-test was performed, unpaired, two-tailed. P-values below 0.05 were considered significant.

### Data availability

The authors declare that the data supporting the findings of this study are available from the authors upon reasonable request.
